# Visualizing the dynamics of histone variants in the S-phase nucleus

**DOI:** 10.1186/s13059-018-1556-4

**Published:** 2018-10-31

**Authors:** Stella Maxouri, Stavros Taraviras, Zoi Lygerou

**Affiliations:** 1 Laboratory of Biology, School of Medicine, University of Patras, Patras, Greece; 2 Laboratory of Physiology, School of Medicine, University of Patras, Patras, Greece

## Abstract

Histone variants constitute a fundamental feature of the epigenome. However, their dynamics during normal and challenged DNA replication and their distribution in the three-dimensional space of the nucleus remain poorly characterized. A recent study employed stochastic optical reconstruction microscopy (STORM) to obtain a high-resolution view of the spatial distribution of H3 histone variants in the nucleus and related this to the timing of DNA replication.

## Introduction

The spatial organization of the genome plays a crucial role in cell function. Histone modifications and histone variants help shape chromatin domains, and this epigenetic landscape governs gene expression and instructs cell function, cellular identity, and fate decisions. However, how histone variants and modifications are distributed in the three-dimensional nuclear volume and how this correlates with nuclear functions remain elusive.

DNA replication poses a major challenge for the epigenome [1]. Duplication of the genome must go hand-in-hand with re-packaging of the newly synthesized DNA into chromatin. Nucleosomes are disassembled ahead of the replication fork and must reform on the daughter strands, preserving the parental epigenetic landscape. Parental histones, bearing their post-translational modifications, are recycled to replicated DNA. As the DNA doubles, however, so must the nucleosomes. Canonical histone genes are specifically expressed in S-phase, to provide material for new nucleosomes in a timely manner. These new nucleosomes must be modified to preserve the epigenome, and this is believed to be dictated by the recycled parental histones. Paralogues of the histone genes, called histone variants, are typically expressed throughout the cell cycle and deposited independently of DNA synthesis [2].

A recent study by the Almouzni laboratory [3] combines genomics with super-resolution imaging of parental histone variants and replication factories to describe the dynamics of histone variants in S-phase at the single-cell level with unparalleled spatio-temporal resolution.

## H3 variants show distinct profiles along the genome

Histone H3 has variants with distinct properties. H3.1 and H3.2 are the canonical replicative variants, deposited by the histone chaperone ‘chromatin assembly factor 1’ (CAF-1) on newly replicated DNA. The H3.3 variant, by contrast, is expressed throughout the cell cycle and is deposited by two different histone chaperones at sites where nucleosomes are displaced, independently of DNA replication. As H3.3 differs in both primary sequence and post-translational modification from H3.1 and H3.2, its distribution along the genome constitutes an epigenetic mark that must be preserved.

In order to assess the genome-wide occupancy of histone H3 variants, Clément and colleagues [3] first performed chromatin immunoprecipitation sequencing (ChIP-seq) and compared histone enrichment profiles against replication timing profiles. They showed that H3.1 and H3.3 were enriched in distinct chromatin domains along the genome. H3.3 was mainly associated with early-replicating domains, in contrast with H3.1 which was enriched in late-replicating regions. H3.3 occupancy was anti-correlated with replication time. Although H3.3 occupancy correlates with transcription, comparison with nascent RNA sequencing data revealed that the association of H3.3 enrichment with replication timing was valid independently of its correlation with the transcriptional profile. This suggests that additional chromatin features, such as accessibility, physical properties, or topology might govern H3.3 deposition.

## A high-resolution view of H3 variant dynamics in the three-dimensional nuclear space

Stochastic optical reconstruction microscopy (STORM) relies on the high-accuracy localization of photo-switchable fluorescent probes in three dimensions to overcome the diffraction barrier of conventional fluorescence microscopy, achieving single-molecule resolution. The authors combined STORM with the SNAP-tag system [4] in order to visualize the dynamics of the H3 variants in cells. By means of this tag, the H3.1 or the H3.3 histone variant was labeled by addition of a fluorescent dye which could then be chased, permitting specific visualization of parental histones bound to chromatin. In parallel, replicating regions were detected at high resolution by incorporation of a nucleotide analogue.

The authors followed the dynamics of total and parental H3.1 and H3.3 histone variants throughout S-phase within the three-dimensional nuclear space. They showed that H3.1 and H3.3 variants form distinct domains inside the nucleus. The high resolution achieved with STORM allowed not only the detailed description of these compartments, but also the detection of changes in size or density by monitoring cells as they progressed through S-phase, revealing the distinct dynamic nature of the H3.1 and H3.3 variants. The authors showed that H3.3 domains are characterized by stable volume throughout the cell cycle and a decreasing density. This is in agreement with the H3.1 variant being deposited by CAF-1 behind the replication fork, leading to dilution of parental H3.3 during replication. By contrast, H3.1 domains exhibit a cell-cycle-dependent profile. In early S phase, H3.1 domains are increased in size and of low density, corresponding to new H3.1 being deposited in H3.3-associated regions during replication. In the rest of the cell cycle, H3.1 units are smaller in size and of high density and correspond to late-replicating chromatin. The distinct distribution of H3.3 and H3.1 within the nucleus highlights the presence of chromatin domains with distinct H3 variant occupancy.

## Replication stress alters the histone variant landscape

Clément and colleagues then investigated how the spatial distribution of histone variants is affected under conditions of replication stress following treatment of cells with hydroxyurea, which depletes deoxynucleotide triphosphate (dNTP) pools. During DNA replication, forks can slow down or arrest, owing to a decrease in nucleotide pools and encountered obstacles such as DNA secondary structure or DNA–RNA hybrids (R-loops). Replication stress is also triggered by oncogene activation and has been suggested to play a key initial step driving carcinogenesis [5]. Following hydroxyurea treatment, local recycling of parental histone variants was severely impaired. Changes in the distribution of parental histones were evident not only at replication sites but also in the surrounding region. This suggests that replication stress might affect the epigenetic landscape by inducing changes in the epigenome that could potentially lead to altered gene expression, thus providing a new potential mechanism for how replication stress might enhance tumorigenesis.

## The histone chaperone ASF1 is essential for preserving the global H3 variant profile

The histone chaperone anti-silencing factor 1 (ASF1) is crucial for histone management. It associates with free H3–H4 dimers to store them when they are in excess and deliver them to CAF-1 or other histone-deposition complexes. It has also been suggested that ASF1 facilitates local delivery of parental histones from the replicative helicase to CAF-1 on the nascent strands. Clément et al. assessed whether ASF1 plays a role in parental histone recycling by silencing ASF1 and found a profound effect on parental histone distribution during replication. The levels of both H3.3 and H3.1 were decreased at replication sites, albeit with different kinetics. Interestingly, when ASF1 was depleted, H3.3 and H3.1 not only decreased on newly replicated DNA but their distribution to distal sites was also affected. Loss of ASF1 therefore not only affects recycling of parental histones but can also alter the histone variant profile throughout the nucleus.

## Concluding remarks

The study from Clément and colleagues has established the distribution of histone H3 variants and their recycling during replication in the three-dimensional space of the nucleus and has linked them with DNA replication timing and gene expression. The authors have shown that H3.1 and H3.3 create domains with distinct characteristics, supporting their distinct functions during DNA replication and transcription. Moreover, the authors observed that disturbing the progression of DNA replication or histone management affects the distribution of parental histones. Parental histone variants that are dissociated from DNA during replication carry their post-translational modifications. Upon replication stress or loss of ASF1 function, the reshuffling of parental histones can cause global epigenetic changes, with effects on chromatin structure and gene expression. This hypothesis is highly interesting in the context of cancer, where replication stress is a common initial event. Intriguingly, impaired recycling of histone variants caused by the absence of the histone chaperone ASF1, independently of replication stress, does not trigger checkpoint activation. This deprives the cells of the opportunity to arrest replication—thus propagating false epigenetic marks and severely challenging epigenomic integrity.

Epigenome stability is crucial for proper cellular function since challenging the propagation of epigenetic marks is closely connected to changes in gene expression. Indeed, a recent study [6] showed that H3.3 is important for maintaining the identity of parental cells during reprogramming. Intriguingly, H3.3 is also essential for the acquisition of pluripotency later in the process of reprogramming. This highlights a central role for H3.3 in cell-fate transitions.

Complementary studies have recently provided further insight into histone dynamics through novel technological advances. For example, a technique known as chromatin occupancy after replication (ChOR-seq) was recently developed to study the occupancy of modified histones on newly synthesized DNA and determine the kinetics of histone recycling upon DNA replication [7]. Reverón-Gómez and colleagues showed that parental histones with their post-translational modifications are accurately recycled during DNA replication, whereas new histones are modified following deposition with varying kinetics. Two other investigations [8, 9] employed techniques permitting assessment of parental histone deposition specifically to the leading and lagging strand during replication. Petryk et al. used mouse embryonic stem cells and showed that minichromosome maintenance protein 2 (MCM2), a subunit of the replicative helicase, facilitates histone recycling to the lagging strand [8]. Yu et al. showed that two non-essential subunits of polymerase epsilon (polε) in budding yeast facilitate histone recycling to the leading strand [9]. These findings raise the intriguing possibility that asymmetric parental histone deposition may be regulated through MCM2 or polε to drive asymmetric fate specification.

These recent studies demonstrate that combining novel methodologies can expand our understanding of how epigenome maintenance is orchestrated in the three-dimensional space to safeguard genomic integrity and instruct pluripotency and cell-fate specification, thus opening a new era of epigenome biology.

## Abbreviations

ASF: Anti-silencing factor
CAF: Chromatin assembly factor
STORM: Stochastic optical reconstruction microscopy
